# Immune Checkpoint Targeted Therapy in Glioma: Status and Hopes

**DOI:** 10.3389/fimmu.2020.578877

**Published:** 2020-11-27

**Authors:** Yangzhi Qi, Baohui Liu, Qian Sun, Xiaoxing Xiong, Qianxue Chen

**Affiliations:** Department of Neurosurgery, Renmin Hospital of Wuhan University, Wuhan, China

**Keywords:** brain immunology, glioma microenvironment, immune checkpoint blockade, immunotherapy resistance, immune-response monitoring biomarker

## Abstract

Glioma is the most malignant primary tumor of the central nervous system and is characterized by an extremely low overall survival. Recent breakthroughs in cancer therapy using immune checkpoint blockade have attracted significant attention. However, despite representing the most promising (immunotherapy) treatment for cancer, the clinical application of immune checkpoint blockade in glioma patients remains challenging due to the “cold phenotype” of glioma and multiple factors inducing resistance, both intrinsic and acquired. Therefore, comprehensive understanding of the tumor microenvironment and the unique immunological status of the brain will be critical for the application of glioma immunotherapy. More sensitive biomarkers to monitor the immune response, as well as combining multiple immunotherapy strategies, may accelerate clinical progress and enable development of effective and safe treatments for glioma patients.

## Introduction

In recent years, novel immunotherapies targeting the immune component of the tumor microenvironment have shown great promise for the clinical management of tumors. Among various therapeutic strategies, drugs targeting immune checkpoint molecules are being heralded as a breakthrough in cancer immunotherapy.

Glioma is the most common and deadliest primary brain tumor of the central nervous system (CNS), with a 5-year survival of less than 10%. Glioblastoma multiforme (GBM) accounts for ~50% of glioma cases and is characterized by a 5-year survival rate of less than 5%, corresponding to a grade IV tumor by the World Health Organization (WHO). Unfortunately, the current gold standard of GBM treatment (total resection plus adjuvant radio-chemotherapy) represents only a palliative option for patients, and the median survival after diagnosis is less than 15 months (1).

A striking recent clinical success of checkpoint inhibitors across multiple solid tumors (2, 3) has sparked interest in immune-targeted strategies for glioma treatment. However, the CNS is commonly considered an “immunologically privileged” site as the blood-brain barrier (BBB) inhibits direct contact between the brain and immune system. Considering the unique accessibility and tissue composition of brain, it is therefore not trivial to design effective immunotherapeutic strategies. Herein, we review the unique immunology and tumor microenvironment of the brain. Furthermore, we describe various immune checkpoint blockade strategies, as well as the mechanisms of resistance to immunotherapy.

## The CNS Is Immunologically “Unique” Rather Than “Privileged”

The term “immunologically privileged” has been commonly used to describe the failure of the brain to reject heterotopic tissue following transplantation in the past decades. Our understanding of this special characteristic of brain immunology largely originates from experiments by Peter Medawar in the 1940s (4). Although allogeneic tissue transplantation in other areas of the body can lead to immune rejection which continues to the CNS, there is a lack of convincing explanations for the fact that this systemic immune state cannot be initiated from the CNS. Medawar attributed this phenomenon to the lack of lymph nodes and lymphatic vessels in the CNS, which result in the perceived absence of efferent information of the CNS, although this view has been recently disproven (5–12). A series of studies have demonstrated that leukocyte lymphatics exist in the dura sinus and transport antigens from the dura to cervical lymph nodes (9–12). These findings propose an interesting mechanism by which cerebrospinal fluid mediates the immune communication between CNS and circulation *via* a glial-lymphatic pathway (5–8). Given the existence of an afferent system between the brain and peripheral immune system, many propose that CNS is immunologically “unique” rather than “privileged.”

For the most part, the BBB is responsible for this “unique immunology” of the brain. Structurally, the BBB consist of a bio-membrane between vascular endothelial cells and glial cells. Functionally, the BBB is a dynamic network between circulation and brain that blocks the diffusion of large, hydrophilic molecules or organisms while allowing the influx of small, hydrophobic molecules (13). Except for a few species, such as Neisseria meningitides and Streptococcus pneumoniae which are able to enter the brain circulation *via* specific mechanisms, the vast majority of blood-borne pathogens are excluded from the brain (14). Given that the CNS is rarely exposed to pathogens, the brain has been believed to only exhibit limited immunity due to poor tolerance of the brain tissue to inflammation. Another unique immunological characteristic of the brain is its resident immune cell population. Originating from myeloid precursors born in the yolk sac, microglial cells (MG) invade the CNS during early embryonic development and serve as the primary resident immune cells (15, 16), while most other immune cell subtypes do not exist in CNS. However, contrary to the previous view that the brain only exhibits limited immunity, recent studies have demonstrated that the systemic immune system is fully involved in the cytotoxic response to CNS antigens (17). After inflammatory stimulation, specific antigens are recognized by MG and presented to activated lymphocytes *via* the glial-lymphatic pathway, after which a large number of immune cells can easily penetrate the BBB, inducing a strong inflammatory and subsequent immune response (18–20). Despite this, both innate inflammatory and adaptive immune responses have to be tightly regulated as unrestrained inflammation-mediated intracranial hypertension can have serious consequences. Although the concept of immunological privilege of the brain has been overturned, the unique immunological environment of the CNS still represents a significant hurdle for therapies targeting immune checkpoints blockade in the brain.

## The Immune Microenvironment of Glioma

**Figure 1:**
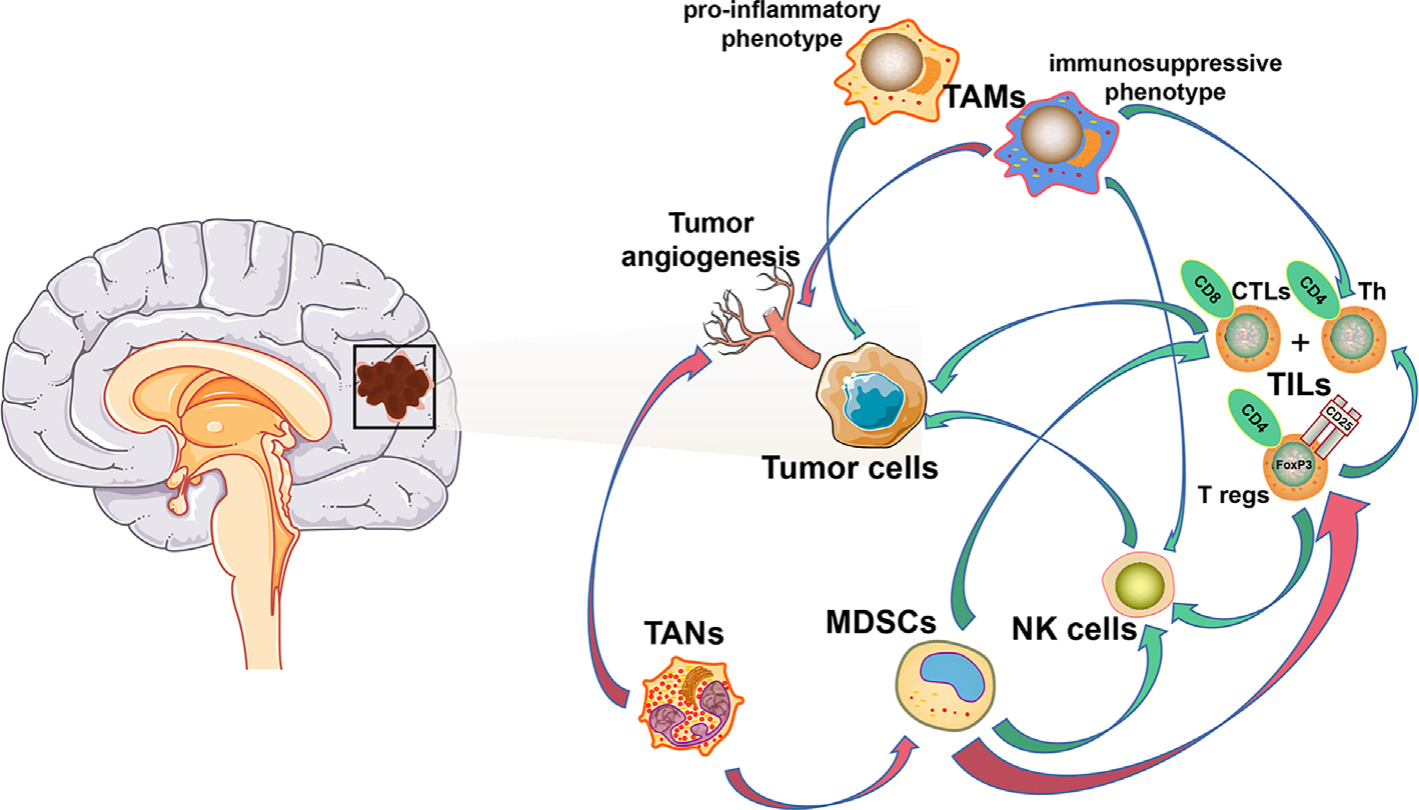
Cellular composition of glioma immune microenvironment. The figure depicts only a general representation of all the cell types that have been reported to be associated with tumor cells in glioma immune microenvironment. Green arrow: down-regulation. Red arrow: up-regulation.

The unique brain immunology leads to a particular tumor microenvironment of glioma. A variety of peripheral immune components are present in this glioma microenvironment, including myeloid derived suppressor cells (MDSCs), natural killer cells (NK cells), macrophages, neutrophils, CD4^+^ helper T cells (Th), CD8^+^ cytotoxic T lymphocytes (CTLs), and regulatory T (T reg) cells (21, 22), while their infiltration ratio is remarkably low numbers in gliomas compared to other tumors. Furthermore, various tumor-derived cytokines and chemokines reprogram infiltrating immune cells, which causes them to acquire unique functional phenotypes and transform into tumor-associated immune cells. These tumor-associated immune cells can therefore have profound effects on progression, recurrence, and therapeutic resistance of glioma by inducing inflammatory or anti-inflammatory responses (**Figure 1**).

### Tumor-Infiltrating Lymphocytes

As the most important component of the immune response in the tumor microenvironment of most solid tumors, tumor-infiltrating lymphocytes (TILs), represented by CD4^+^ Th, CD8^+^ CTLs, and CD4^+^/CD25^+^/FoxP3^+^ T reg (23–26), are only present in remarkably low numbers in the CNS compared to other tumor types. CD4^+^ Th and CD8^+^ CTL populations increase with tumor malignancy, starting at 39% in WHO grade II tumors, rising to 73% in WHO grade III, and 98% in grade IV (22). Meanwhile, a correlation between increased CD8^+^ CTL counts and improved patient outcomes has previously been reported (27). T reg cells have a suppressive role in the adaptive immune response and inhibit the proliferation of Th cells and CTLs by secretion of suppressive cytokines (26).

The limited activity and number of TILs in the brain is predominantly caused by the unique immunological status of the brain which encourages only limited immunity in order to prevent an inflammation-mediated intracranial hypertension crisis. In response to tumor-derived inflammatory stimulation, T reg cells secrete anti-inflammatory interleukin-10 (IL-10) and transforming growth factor β (TGF-β) in order to dampen an inflammatory immune response against the tumor (28, 29). In addition to immunosuppressive mechanisms of the CNS, expression of certain genes by the tumor itself also contributes to low levels of TILs. For instance, glioma cells produce a high level of indolamine 2, 3-dioxygenase (IDO) which activates suppressor T cells by depleting tryptophan from the tumor microenvironment (30). Besides, tumor-derived Fas ligand promotes apoptosis of activated T cells and leads to an immune escape of tumor cells by inhibiting dendritic cells and maturation of T cells (31). Overexpression of programmed cell death-ligand 1 (PD-L1) in glioma cells prevents activation of T cells and induces T cell apoptosis *via* binding to programmed death 1 (PD-1), a well-known inhibitory immune checkpoint molecule (32, 33). Moreover, an overexpression of CTLA-4 mRNA and protein, a strong CD4^+^ T cell and CD8^+^ CTL inhibitor, is caused by lack of CD80/86 co-stimulatory molecules (22). Therefore, a comprehensive understanding of tumor heterogeneity and the role of T cells in glioma is of critical importance for the design of therapeutic targets.

### Tumor-Associated Macrophages

Tumor-associated macrophages (TAMs) are the major infiltrating immune component in the glioma microenvironment, accounting for ~50% of all immune cells, and have an important role in neoplasia, metastasis, immune escape, and tumor angiogenesis (34, 35). Several studies have reported that a majority of TAMs are derived from circulating monocyte-derived macrophages (MDMs), while the remaining proportion originates from MG (36–38). Immature monocytes migrate to the tumor microenvironment and develop into TAMs following exposure to several cytokines (34, 35). In the glioma microenvironment, tumor- or effector T cell-derived cytokines promote a change in macrophage effector mechanisms on a spectrum between a pro-inflammatory “M1” phenotype with anti-tumor responses, and an immunosuppressive “M2” phenotype with anti-inflammatory responses (39). In the early stages of glioma, TAMs inhibit tumor proliferation *via* the pro-inflammatory “M1” phenotype, while in advanced glioma, TAMs are predominantly characterized by the “M2” phenotype, which generally induces an immunosuppressive response and immune escape of the tumor. As a special type of TAMs in CNS, MG also exhibit similar plasticity to monocyte-derived macrophages (40).

Studies have demonstrated that large numbers of infiltrating TAMs are closely associated with poor prognosis (41) and the “M2” phenotype has been shown to promote tumor progression *via* secretion of immunosuppressive cytokines and factors promoting angiogenesis (41, 42). Given this evidence, it seems feasible to block the formation and phenotypic “M2” transformation of TAMs. In mouse models, CSF-1 receptor inhibition with small molecules either blocks the transformation of “M2” phenotype or depletes TAMs (43–46), both of which inhibit glioma progression and invasion. Meanwhile, some other drugs have also been shown to achieve their anti-tumor effect by depleting monocytes that serve as precursors of TAM (47). However, recent studies have expanded our understanding of macrophage polarization (48) and revealed a multifaceted response comprising classical M1 and M2 polarization, including expression changes associated with chronic inflammatory stimuli and exposure to free fatty acids, which is involved in regulation of bone marrow cell function. This indicates that the diverse transcriptional programming of TAMs in glioma extends beyond the simplified view of an “M1” versus “M2” polarization. Thus, despite the fact that both depletion of TAMs and targeting “M2” polarization can represent attractive therapeutic approaches for glioma, a more comprehensive understanding of TAM phenotypes is required for efficient and safe treatments of glioma (43, 49, 50).

### Myeloid-Derived Suppressor Cells

Chronic inflammation in the tumor microenvironment is induced by overexpression of pro-inflammatory cytokines, including CSF-1, VEGF, TGF-β, and tumor necrosis factor α (TNF-α) (51, 52). These pro-inflammatory cytokines promote tumor growth, progression, and resistance to immunotherapy by inducing a transformation of immature myeloid cells into myeloid-derived suppressor cells (MDSCs). MDSCs are recruited to peripheral lymphoid organs and the tumor microenvironment from the bone marrow, promoting tumor cell proliferation *via* various mechanisms, including suppression of cytotoxic NK cell activity, inhibition of the adaptive T cell response, induction of T cell apoptosis and T reg cell proliferation, and secretion of immunosuppressive cytokines (53–56). Therefore, MDSCs contribute to resistance to immunotherapy, and combining treatments targeting MDSCs with other immunotherapies has become a promising therapeutic strategy achieving considerable success (57–61). In glioma, related research has been focusing on strategies that either inhibit the recruitment (targeting of C-C motif chemokine ligand 2, VEGF-A, IL-8, and galectin-1) or the formation of MDSCs from myeloid precursors (targeting of M-CSF, PI3Kγ, TAM-RTKs, and COX-2). Such strategies have shown great promise in preclinical studies (62). As there is increasing evidence that the function of MDSCs is tumor type-dependent, a clear definition of this cell type in glioma remains warranted (49). Transcriptomic characterizations of MDSCs - separately from MG and MDMs – should be carried out to ascertain the suppressive function and mechanisms of differentiation into MDSCs, which could help to evaluate the clinical value of MDSCs-targeted therapies in glioma (63).

### Tumor-Associated Neutrophils

Completely contrary to their pro-inflammatory function during infections, neutrophils have been frequently reported to promote tumor progression and metastasis in recent years (64–66). This unique relationship between neutrophils and tumor cells could provide a reasonable explanation for the phenomenon that circulating tumor cells often escape from immune surveillance in breast cancer as neutrophils account for the largest proportion of circulatory leukocytes (66). Besides, current study also indicated that immunosuppressive tumor-associated neutrophils (TANs) or granulocytic MDSCs are enriched in neutrophil-enriched subtypes of triple negative breast cancer and were associated with acquired immune checkpoint blockade resistance (67). In the glioma microenvironment, TANs promote tumor malignancy by mediating angiogenesis (68). Besides, TAN depletion strategies using a Ly6G^+^ monoclonal antibody have been shown to prolong overall survival in preclinical GBM mouse models (69). However, the mechanisms underlying TAN recruitment to the tumor microenvironment and the role of TANs in tumor progression are not yet comprehensively understood and how the glioma microenvironment heterogeneity affects neutrophil reprogramming still remains to be unraveled.

### Natural Killer Cells

A variety of mechanisms suppressing the activity of natural killer (NK) cells, the most efficient innate cytotoxic lymphocytes, have been identified during tumor cell progression. Similar to normal cells, glioma cells can inhibit antigen presenting cell (APC)-mediated recognition and NK cell-mediated killing through expression of MHC class I molecules (MHC I) that interact with NK cell immunoglobulin-like receptors (KIRs) (49). Besides, infiltrating NK cells in the glioma microenvironment have been reported to be commonly nonfunctional, largely owing to the combined negative regulatory effect of TAMs, MDSCs, and T reg cells (49, 70).

## Immune Checkpoint Blockade Strategy and Inhibitors

There is no doubt that among various immunotherapies, despite that checkpoint blockade might not be the most promising treatment for glioma, it has been the immunotherapy most developed in clinical use. Via a combination of specific antibodies and checkpoint molecules, effector T cells can be reactivated and exert tumor cell cytotoxicity. In the next paragraphs, we describe classical immune checkpoint molecules and their inhibitors (**Table 1**).

**Table 1:** Current clinical trials of immune checkpoint blockade.

Clinical trials	Stage	Targets	Drugs
Monotherapy			
NCT02017717 (CheckMate⁃143)	III	PD-1	Nivolumab
NCT02617589 (CheckMate⁃498)	III	PD-1	Nivolumab + radiation
NCT02667587 (CheckMate⁃548)	III	PD-1	Nivolumab + radiation + TMZ
NCT02648633	III	PD-1	Nivolumab
NCT03718767	II	PD-1	Nivolumab
NCT03797326	II	PD-1	Pembrolizumab
NCT02852655	II	PD-1	Pembrolizumab
NCT02337686	II	PD-1	Pembrolizumab
NCT02968940	II	PD-L1	Avelumab + radiation
NCT03047473	II	PD-L1	Avelumab + TMZ
NCT03341806	I	PD-L1	Avelumab
Combined with other checkpoint molecules			
NCT03707457	I	PD-1+IDO1	Nivolumab + INCB024360
NCT04047706	I	PD-1+IDO1	Nivolumab + BMS986205
NCT02658981	I	PD-1+LAG-3	Nivolumab + BMS986016
NCT03493932	I	PD-1+LAG-3	Nivolumab + BMS986016
NCT03233152	I	PD-1+CTLA-4	Nivolumab + Ipilimumab
NCT03422094	I	PD-1+CTLA-4	Nivolumab + Ipilimumab
NCT02311920	I	PD-1+CTLA-4	Nivolumab + Ipilimumab+TMZ
NCT02794883	II	PD-L1+CTLA-4	Durvalumab + Tremelimumab
Combined with VEGF/VEGFR			
NCT03743662	II	PD-1+VEGF	Nivolumab + BEV + radiation
NCT02336165	II	PD-L1+VEGF	Durvalumab + BEV
NCT03291314	I	PD-L1+VEGFR	Avelumab + Axitinib
NCT02052648	I/II	IDO1+VEGF	Indoximod + BEV + TMZ
Combined with CAR-T			
NCT03726515	I	PD-1+CAR-T	Pembrolizumab + CAR⁃EGFR-III⁃T
NCT04003649	I	PD-1+CTLA-4+CAR-T	Nivolumab + Ipilimumab + CAR-T
Combined with vaccines			
NCT02529072	I	PD-1	Nivolumab + DC vaccines
NCT02287428	I	PD-1	Pembrolizumab + NeoVax vaccines
NCT03750071	I/II	PD-L1	Avelumab + VXM01 vaccines

PD-1, programmed cell death protein 1; PD-L1, programmed cell death 1 ligand 1; IDO1, indoleamine 2,3-dioxygenase; LAG-3, lymphocyte-activation gene 3; CTLA-4, cytotoxic T-lymphocyte associated protein 4; VEGF, vascular endothelial growth factor; VEGFR, vascular endothelial growth factor receptor; CAR-T, chimeric antigen receptor T-cell; TMZ, temozolomide.

### PD-1/PD-L1

PD-1 and its ligands PD-L1/2 are the most comprehensively studied immune checkpoint molecules to date. PD-1 negatively regulates T cell receptor-mediated signaling transduction pathways and, in combination with PD-L1, inhibits activation and cytotoxic T cell effects and blocks the production of inflammatory factors, resulting in T cell inactivity. Expression of PD-1 on immune cells is tightly regulated. For instance, PD-1 expression appears on the surface of T cells shortly (less than 24 h) after T cell activation and decreases with the elimination or clearance of the antigen (49). Under chronic inflammatory conditions or in cancer, antigens repetitively stimulate CTLs to maintain high levels of PD-1 expression, eventually resulting in T cell exhaustion and depletion. Tumor-expressed PD-L1 is regulated by several mechanisms, including phosphatidylinositol 3-kinase (PI3K) signaling pathway activation and TIL-secreted interferon γ (IFN-γ) (71). In glioma, PD-L1 is predominantly expressed on tumor cells and TAMs and negatively relates to patient outcome (72–74). To date, two anti-PD-1 antibodies (Nivolumab, Pembrolizumab) and three anti-PD-L1 antibodies (Atezolizumab, Avelumab, Durvalumab) have been put into clinical application and have achieved dramatic successes against a variety of solid tumors (75–77). However, they have so far not been approved for clinical treatment of GBM despite numerous preclinical successes reported over the past decades (78–83). For instance, in the preclinical GL261 model, anti-PD-1 treatment success is dosage dependent, with the best outcome reported being a cure rate of 50% (81, 83). Anti-PD-1 monotherapy has been observed to result in an increased ratio of CD8^+^ CTLs to T reg cells, and enhanced efficacy when combined with radiation and other checkpoint inhibitors (81, 83).

Schalper et al. (84) reported treatment of 30 GBM patients (3 primary, 27 recurrent) with preoperative and postoperative nivolumab (NCT02550249), resulting in increased transcription of chemokines, infiltration of TILs, and diversity of TCR in tumor microenvironment. While no patients with recurrent GBM benefited from treatment as measured by overall survival (OS), two of the three primary GBM patients survived for 33 month and 28 months, respectively. CheckMate⁃143 phase III trial (NCT02017717) found no OS benefit when comparing nivolumab with bevacizumab (anti-VEGFA) in the treatment of recurrent GBM (median OS 9.8 vs. 10.0 months) (85). In the CheckMate⁃498 trial (NCT02617589), newly diagnosed GBM patients with unmethylated O6-methylguanine-DNA methytransferase (MGMT) promoter who received nivolumab plus radiotherapy did not benefit from this treatment compared with radiotherapy plus temozolomide (TMZ) as measured by OS (86). More recently, similarly disappointing results have been reported in the CheckMate-548 study (NCT02667587). Here, newly diagnosed GBM patients with methylated MGMT promoter did not show a PFS benefit with anti-PD-1 treatment; the OS effect is still pending (87). Primary results of a study by Lukas et al. (88) reporting on a clinical trial using atezolizumab, an anti-PD-L1 antibody (NCT01375842), showed that increased CD4^+^ T cells and IDH mutation indicated better treatment efficiency of atezolizumab.

### CTLA-4

Cytotoxic T-lymphocyte associated protein 4 (CTLA-4) expression on activated T cells or T reg cells was the first identified member of the immunoglobulin superfamily, and also the first immune regulation molecule used in targeted therapy. CTLA-4 inhibits T cell co-stimulatory signaling pathways by combining with ligands CD80 and CD86 expressed on APCs (89). Unlike PD-1, CTLA-4 signaling occurs at the early stages of T cell activation, and CTLA-4 is mainly expressed on T cells of the lymph node (90). In preclinical experiments, anti-CTLA-4 monotherapy prolonged OS in the GL261 syngeneic mouse model (81). Although CTLA-4 blockade strategy results in an increased median survival with 25% cure rate, the response of monotherapy was still considered limited as combined application of anti-PD-1 therapy or radiotherapy can remarkably improve efficacy (81, 90). Reardon et al. (81) also reported that combination of anti-CTLA-4 and anti-PD-1 therapy increased the cure rate to 75%. For further investigation, CTLA-4 blockade as a monotherapy or in combination with anti-PD-1 treatment is therefore currently being tested in a phase III clinical trial in patients with recurrent GBM (NCT02017717).

### B7 Family

In recent years, there have been increasing numbers of studies investigating immune checkpoint molecules of the B7 family. In addition to PD-L1 (B7-H1), studies have investigated B7-H3 (CD276), B7-H4, B7-H5 (Vista), B7-H6, and B7-H7 (HHLA2), amongst others. B7-H3 and B7-H7 have a dual function, enabling both co-stimulation and co-inhibition (91). By interacting with specific ligands, these molecules can therefore have different roles in tumor progression. For instance, recent research points out that B7-H3 positively relates to the Toll-like receptor signaling pathway and the poor survival of glioma patients, while it has also been reported to co-stimulate immunological function and be involved in anti-tumor functions (92–95). Similarly, B7-H7 shows the same phenomenon in various solid tumors (96, 97). Inhibiting NK-mediated recognition of B7-H6 is an important mechanism of the tumor immune escape. NK cells eliminate B7-H6-expressing tumor cells either directly *via* cytotoxicity or indirectly by cytokine secretion, which highlights a role for the tumor-induced “self”-molecule B7-H6 in alerting innate immunity (91). Both B7-H4 and B7-H5 have co-inhibitory functions on the immune system (91), although research on these and other members of the B7 family is still in progress. As the largest immune checkpoint family, the function and mechanisms of B7 family members in glioma remains largely unknown. Thus, a more comprehensive understating of the function of the B7 family in glioma could help to explore more effective therapeutic targets in immunotherapy.

### IDO, LAG-3, and TIM-3

Indoleamine 2,3-dioxygenase (IDO) is the key enzyme of the L-tryptophan metabolism *via* the kynurenine pathway. Although IDO expressed on tumor cells and dendritic cells (DCs) is not a typical checkpoint molecule, it can inhibit T cell activation by modulation of the tryptophan metabolism which has an important role in the function of T cells (98–101). Metabolites of tryptophan also induce T cell apoptosis (101). Besides, an interaction of kynurenine and TGF-β can induce FoxP3 expression in T cells, which results in T reg cell polarization (102, 103). Preclinical models have shown that clinical trials with IDO inhibitors did not meet the expectations (104).

Lymphocyte-activation gene 3 (LAG-3) has four extracellular immunoglobulin superfamily-like domains which bind to MHC II, and is responsible for transmission of inhibitory signals (105). In addition to MHC II, another ligand for LAG-3 is Gal⁃3, which is involved in the inhibition of CD8^+^ CTLs (106). Tumor-derived antigens induce LAG-3 overexpression and thereby lead to the depletion of CD8^+^ CTLs (107). Research in mouse xenografts revealed that co-targeting of PD-1 and LAG-3 on TILs can limit tumor growth, which is likely superior to a single inhibitory mechanism (108, 109). Given this finding, recent trials have focused on anti-PD-1 and anti-LAG-3 combination therapies rather than monotherapies. However, the vast majority of this research in still in preclinical stages.

T cell immunoglobulin domain and mucin domain protein-3 (TIM-3) is expressed on CD4^+^ and CD8^+^ T cells, monocytes, and macrophages (110). TIM-3 regulates T cell depletion and is involved in tumor immunosuppression and immune escape *via* binding to its ligand Gal-9 (110). Clinical trials reported that GBM patients with overexpression of TIM-3 have higher tumor malignancy, a lower quality of life, and worse prognosis (111, 112).

Although several checkpoint-related molecules have been discovered, there have been none as influential as PD-1 and CTLA-4, and the efficiency of the vast majority of checkpoint inhibitors in glioma remains doubtful. While single checkpoint inhibition is the standard of care in many tumor entities, checkpoint molecules cooperate or antagonize each other in tumor progression, making it difficult for a single checkpoint inhibitor to play a decisive role in systemic immunity. Therefore, combination of checkpoint inhibitors seems to be more efficient than monotherapy.

## Lessons From Clinical Failures

There is no doubt that immunotherapy holds promise for the treatment of glioma. However, even promising preclinical data are rarely translated into clinical success in glioma. Two factors complicate the clinical translation for glioma treatment. Firstly, glioma has a “cold tumor” phenotype, which is associated with a poor response to immunotherapy. Owing to the unique environment of CNS, even after inhibiting checkpoint molecules to induce T cell responses against glioma, antigen-specific TILs remain at relatively low levels. Second, current preclinical models have only limited capacity to reflect the real tumor heterogeneity of glioma. Generally, GBM can be classified into four subtypes: classical, proneural, neural, and mesenchymal, with high heterogeneity between each subtype (113, 114). There are remarkable differences in gene expression among these four subtypes, which suggests that targeting checkpoint molecules therapies may only be effective for some subpopulations expressing specific genes, but not for other subpopulations. These two factors interact to form resistance mechanisms at all phases of the antitumor immune response: intrinsic resistance prevents the initiation of a response; adaptive resistance deactivates tumor-infiltrating immune cells; and acquired resistance protects the tumor from elimination in the face of attack by the immune system. Even though dramatic immune responses have been observed in preclinical models using a variety of immunotherapy strategies, patients rarely benefit from these treatments, owing to the extensive immunosuppressive mechanisms of glioma (115, 116). However, these mechanisms render glioma a valuable model for studying how resistance allows tumors to escape immunotherapy.

### Intrinsic Resistance

Intrinsic tumor resistance can be classified into three groups: patient-intrinsic factors (including sex, age, and HLA genotype), tumor-intrinsic factors (including the host immune system and tumor-associated stroma), and environmental factors (117–119). Among them, tumor-intrinsic factors, relating to the genetic, transcriptional or functional profile of the tumor cells, are the main determinants of response and resistance (116).

Several studies have demonstrated that tumors can prevent immune responses by not expressing high-quality neoantigens, and they can furthermore rapidly suppress immune responses by expressing multiple immune checkpoint ligands and immunosuppressive cytokines (115, 116). Meanwhile, even with sufficient antigenicity, sensitivity to immune checkpoint blockade can be disrupted by tumor­intrinsic genetic defects in the IFN γ signaling pathway and antigen presentation (120–122). A disruption in anti-tumor response to the IFN γ signaling pathway can inhibit the Janus kinase (JAK) and (STAT) signaling pathway, downregulating PD-L1 expression, and making anti-PD-1 treatment ineffective. Besides, the WNT–β-catenin signaling pathway has been confirmed to prevent an anti-tumor immune response by inhibiting dendritic cells and promoting the immunosuppressive cytokine IL-10 (123–126). Meanwhile, the MAPK signaling pathway also contributes to tumor immune escape by upregulation of the expression of the immunosuppressive cytokines IL­6 and IL­10 (127).

To date, the heterogeneity of glioma is still considered the basis for its resistance to a variety of treatments. For instance, the most extensively studied neoantigen, EGFR variant III, is a truncated EGFR neoantigen with expression in 19% of newly diagnosed GBM patients, of which 11% exhibit high levels of expression (128). Although nearly 82% of recurrent tumors do not express EGFR variant III, the vast majority of mesenchymal subtypes shows overexpression of EGFR variant III (129, 130). This characteristic makes it difficult to stably express specific antigens to induce a durable anti-tumor immune response. Besides, despite the fact that adjuvant radio-chemotherapy can enhance the efficiency of checkpoint blockade strategies, what cannot be ignored is that radio-chemotherapy has well-­documented immunosuppressive functions inducing other resistance mechanisms rather than tumor-intrinsic resistance to immunotherapy, which further reduces the immune responses of the CNS (131).

### Adaptive Resistance

The discovery that tumors can counter attacks of the immune system by usurping mechanisms that normally prevent autoimmunity is one of the most impactful findings in the history of oncology. Although immune checkpoint molecules may be expressed in various tumors at “baseline,” a remarkable increase of their expression levels can be observed under immunological stimulation (132). Thus, immune checkpoint blockade can trigger strong anti-tumor response. In spite of the durable clinical responses that PD-1 and CTLA-4 blockade strategies have achieved in several advanced tumors, it is undeniable a large proportion of patients do not benefit from checkpoint blockade (132). One explanation for this is that TILs can exhibit severe exhaustion, similar to that observed in chronic viral infections (133). However, while the degree of immune exhaustion in GBM is severe, it does not appear to be singularly so, as other tumors that respond poorly to checkpoint inhibitors use similar adaptive resistance mechanisms (115, 134). Another explanation is that checkpoint molecules with similar mechanisms can compensate for each other. For instance, upregulation of the alternative checkpoint molecule TIM-3 has been observed in tumors resisting PD-1 blockade (135). Downregulation of one immune checkpoint generally upregulates alternative immune checkpoints, eventually leading to the durable immunosuppression and a resistance to the blockade. Given this mechanism, current clinical trials focus on overcoming adaptive resistance of PD-1 and CTLA-4 blockade strategies by targeting alternative immune checkpoints.

### Acquired Resistance

Acquired resistance of tumor generally refers to the genetic alternations caused by immunological pressure (115). For instance, in non-small cell lung cancer (NSCLCs) and melanoma, significant downregulation of targeted antigens has been observed in tumor infiltrating region, resulting in the failure of immune targeted therapy (136, 137). Perhaps therapies that overcome intrinsic resistance mechanisms will also render acquired resistance inconsequential by generating a diverse repertoire of T cell clones targeting high-quality targeted antigens that rapidly eliminate a tumor before acquired resistance emerges. However, the exact effects of acquired resistance on malignant glioma remain unknown, as the low response and persistence of treatments in glioma have been considered as an important intrinsic resistance mechanism. In contrast, recent research reported 66 recurrent GBM patients who received PD-1 blockade therapy (138). Among them, 17 patients were identified as responders based on brain imaging and profiling of resected tissue. Tumors in responders were found to be enriched for alterations in the mitogen-activated protein kinase pathway and exhibited branched patterns of evolution, while non-responding tumors more frequently had mutations in the gene encoding PTEN and non-clonal evolution patterns (138). Notably, responders had a significantly longer OS than non-responders (14.3 vs. 10.1 months) (138). Given the heterogeneity of GBM mentioned above, in addition to intrinsic resistance, acquired resistance seems to play an important role in resistance to checkpoint blockade.

## Hypoxia in the Glioblastoma Microenvironment

To date, research has mainly focused on the “seed’s” response to therapy (i.e., tumor cells themselves), while the problem of “poor soil” (the tumor microenvironment) is often ignored. Herein, we further explored the role of hypoxia in the tumor immune microenvironment. Accumulating evidence indicates that hypoxia may protect tumors from immune responses through various mechanisms, including by inhibition of NK and CTL cell activity, promotion of immunosuppressive cytokines, and by enhancing immunosuppressive cells (T reg cells, TAMs, and neutrophils) (139).

### CTLs and NK Cells

There are an increasing number of studies investigating the effects hypoxia on immune cells. For instance, IL-2, an important growth factor for T and NK cells with a pivotal role in the regulation of the host’s immune response, has been reported to be exquisitely sensitive to changes in oxygen tension (140). Hypoxia can cause a prolonged reduction in IL-2 mRNA expression and inhibit NK cell and CTL activity. Meanwhile, hypoxia has also been shown to reduce the ability of NK cells to release IFNγ, TNFα, GM-CSF, CCL3, and CCL5 (139, 141). In patients with a high risk of hypoxia, CTLs and NK cells appeared to be in resting status rather than active (139), revealing that hypoxia might lead to a state of immune suppression.

### Suppressive Immune Cells and Cytokines

Hypoxia is thought to play a key role in TAM polarization. It can promote the “M2” phenotype and contribute to tumor growth, immune suppression, and tumor angiogenesis (142–144). In a bioinformatic study assessing the polarization of cells in the tumor immune microenvironment, T reg cells, neutrophils, and TAMs with an “M2” phenotype increased remarkably under hypoxia (139). Besides, hypoxia also promotes the expression of TGF-β and IL-10, two well-established suppressive cytokines (139, 142, 145).

## Future Directions

The extensive immunosuppressive mechanisms in “seed” (including tumor heterogeneity and alteration of checkpoint molecules) and “soil” (hypoxia in tumor microenvironment) complicates the treatment of glioma and explains why promising preclinical data had rarely been translated into clinical success. Given this, individualized treatment and real-time monitoring of treatment response are essential.

### Biomarkers

Predicting and monitoring patient responses to treatment have become an urgent requirement for the clinical development of immunotherapies. Tumor tissue biopsies remain the gold standard for diagnosis, but its application is not suitable for response monitoring. Complex and changeable signals on MRI furthermore challenge the differentiation of glioma recurrence from pseudoprogression and radiation brain necrosis. Thus, the availability of biomarkers has greatly enhanced oncological practices and is now the basis of precision medicine for many cancers. However, suitable biomarkers for immunotherapies of glioma are still unknown. Recently, studies have reported that anti-PD-1 therapy results in upregulation of T cell- and IFN γ-related gene expression in immune cells, as well as downregulation of cell-cycle-related gene expression within tumor cells (84, 138, 146). Anti-PD-1 therapy seems to result in different responses in tumors with specific genetic alternation, including increased clonal expansion of T cells, decreased expression of PD-1 in peripheral T cells, and decreased monocytes in circulation (84, 138, 146). Liquid biopsies are anticipated to become a successful strategy for biomarker response monitoring in glioma. For instance, tumor mutation burden (TMB) based on detection of circulating tumor DNA shows a high correlation with anti PD-1 response (147). Meanwhile, current studies also indicated that circulating tumor cells (CTCs) of glioma offer unique advantages for non-invasive monitoring of tumor progression which could furthermore identify pseudoprogression and radiation necrosis (148, 149). Taken together, an efficient biomarker can not only help to choose individualized treatment, but also timely reflect when patients develop resistance to adjust the treatment.

### Combined Drug Therapy

Immunotherapy resistance of glioma is a result of multiple factors: intrinsic resistance and adaptive resistance in the early stages of treatment, and acquired resistance over the period of therapy mediated by genetic alternations. Owing to unique resistance mechanisms, monotherapy of checkpoint inhibitors for glioma does not seem to induce durable anti-tumor responses. Thus, combined drug therapy, to some extent, may show advantages and higher efficacy. For instance, in preclinical model, anti-PD-1 combined with anti-TIM-3 synergistically improved survival (135). Furthermore, the combination of immune checkpoint blockade and anti-tumor-associated immune cells (TAMs, MDSCs) also holds promise for the treatment of glioma. Therefore, a more comprehensive understanding of immune cell roles in the tumor microenvironment, as well as specific biomarkers for functional immune cell types and tumor response, may be necessary for individualized treatment of patients with glioma in the era of precision medicine.

## Author Contributions

YQ and BL contributed equally to this article. XX and QC designed this study. YQ, QS and BL performed the data collection and collation. All the authors were involved in the analysis and interpretation of data. YQ wrote the paper, with the help of the coauthors. XX and QC reviewed and revised the manuscript. All authors contributed to the article and approved the submitted version.

## Conflict of Interest

The authors declare that the research was conducted in the absence of any commercial or financial relationships that could be construed as a potential conflict of interest.
